# Chronic pulmonary aspergillosis is common among patients with presumed tuberculosis relapse in Ghana

**DOI:** 10.1093/mmy/myac063

**Published:** 2022-08-11

**Authors:** Bright K Ocansey, Benjamin Otoo, Abraham Adjei, Hafisatu Gbadamosi, Fleischer C N Kotey, Chris Kosmidis, Jane S Afriyie-Mensah, David W Denning, Japheth A Opintan

**Affiliations:** Division of Evolution, Infection and Genomics, Faculty of Biology, Medicine and Health, University of Manchester, Manchester Academic Health Science Centre, Manchester, M13 9NT, UK; Department of Bacteriology, Noguchi Memorial Institute of Medical Research, University of Ghana, Legon, GA-337, Ghana; Chest Diseases Unit, Department of Medicine and Therapeutics, Korle-Bu Teaching Hospital, Accra, GA-221, Ghana; Radiology Department, Korle-Bu Teaching Hospital, Accra, GA-221, Ghana; FleRhoLife Research Consult, Accra, GZ-077, Ghana; Department of Medical Microbiology, University of Ghana Medical School, Accra, GA-270, Ghana; Division of Evolution, Infection and Genomics, Faculty of Biology, Medicine and Health, University of Manchester, Manchester Academic Health Science Centre, Manchester, M13 9NT, UK; National Aspergillosis Centre, Manchester University NHS Foundation Trust, Manchester, M23 9LT, UK; Chest Diseases Unit, Department of Medicine and Therapeutics, Korle-Bu Teaching Hospital, Accra, GA-221, Ghana; Department of Medicine and Therapeutics, University of Ghana Medical School, Accra, GA-221, Ghana; Division of Evolution, Infection and Genomics, Faculty of Biology, Medicine and Health, University of Manchester, Manchester Academic Health Science Centre, Manchester, M13 9NT, UK; Department of Medical Microbiology, University of Ghana Medical School, Accra, GA-270, Ghana

**Keywords:** Aspergillus serology, chronic pulmonary aspergillosis, Ghana, relapse, tuberculosis

## Abstract

Chronic pulmonary aspergillosis (CPA) may mimic pulmonary tuberculosis (PTB). The two diseases are clinically indistinguishable and may result in CPA misdiagnosed as PTB or vice versa. Although PTB is largely recognised as a differential diagnosis of CPA and often ruled out prior to CPA diagnosis, the reverse is uncommon. The aim of this study was to determine the proportion of CPA cases among patients being assessed for PTB. A cross-sectional survey was conducted among consecutive patients referred for GeneXpert *Mycobacterium tuberculosis* test for the diagnosis of PTB at the Korle-Bu Teaching Hospital, Accra, Ghana. Patients’ demographics, clinical and socioeconomic details were obtained using a structured questionnaire. Blood was collected for *Aspergillus* and HIV serology, and sputum samples obtained for *Aspergillus* culture. Chest radiograph was obtained, and computed tomography scan was also done for patients with positive *Aspergillus* serology or cavitation. CPA was defined using an algorithm developed by the Global Action for Fungal Infections (GAFFI) international expert panel. A total of 154 patients were included in the analysis, of whom 134 (87%) did not have a prior PTB diagnosis. There were 41 (26.6%) GeneXpert positive cases. CPA prevalence was 9.7% overall, but 50% in patients with a prior history of PTB and 3.7% in those without previous PTB. Although CPA is rarely considered as a differential diagnosis of PTB in Ghana, our findings show that CPA may affect half of patients being assessed for PTB relapse. Efforts to diagnose CPA should be prioritised in this patient group.

## Introduction

Pulmonary fungal infections have increased in clinical significance in recent times, and although many of them mimic pulmonary tuberculosis (PTB), chronic pulmonary aspergillosis (CPA) is one of the most common.^1^ CPA is a slow, progressive, and destructive lung disease associated with both respiratory and systemic symptoms. Globally, approximately 3 million people suffer from CPA, with 1.2 million occurring as a sequel of PTB.^2^ In Ghana, CPA among PTB patients is estimated at 2600 cases annually.^3^ PTB is a common differential diagnosis of CPA, and could occur before, after, or infrequently, together with CPA.^4^ There are many similarities between PTB and CPA in terms of risk factors, clinical presentation, and radiological features, making the two diseases clinically indistinguishable.^5^ This may result in misdiagnosis of CPA as PTB, or vice versa. As PTB is more common and largely recognised globally, the index of suspicion for PTB is likely higher compared to CPA, particularly in settings with a high PTB burden. Being mostly diagnosed as a post-PTB complication, CPA may be misdiagnosed as relapsed PTB infection and managed as such.^5^ CPA may also be occasionally misdiagnosed as primary TB infection.^5^ Some studies have reported CPA misdiagnosed as acid-fast bacilli (AFB) smear-negative or GeneXpert *Mycobacterium tuberculosis* (MTB)-negative PTB and resulting in worsening symptoms and anti-TB treatment failure.^6,7^ Previous and present guidelines for CPA diagnosis have recommended a necessary exclusion of PTB.^4,8^ However, with emerging concerns of primary CPA and CPA co-existing with PTB, it may be equally important to rule out CPA when making a diagnosis of PTB to avoid inappropriate exposure of patients to anti-TB medications.

Unfortunately, differentiating CPA from PTB in many high TB burden countries, which are mostly resource-constrained, is a major challenge. This is probably because of inadequate awareness, unavailable diagnostic laboratory support, and omission of CPA as a differential diagnosis in existing local guidelines. To improve the *status quo*, the Global Action for Fungal Infections (GAFFI) convened an international expert panel in 2016 to develop a CPA guideline specific for resource-constrained settings.^8^ However, the lack of *Aspergillus* serology testing capacity, which is key to CPA diagnosis, poses a significant limitation to the general use of these guidelines. ^9–11^ Until recently, the common standard and commercially available methods were precipitins and enzyme immunoassay. The drawbacks of these techniques include cost, long turnaround time, poor inter-laboratory reproducibility, and variable cut-off values.^9,12–14^ Additionally, these tests require sophisticated equipment and adequate laboratory expertise. LDBio Diagnostics introduced a new rapid diagnostic test (RDT) in the form of a lateral flow assay (LFA) for the detection of *Aspergillus*-specific IgG and IgM antibodies based on immunochromatography technology that meets the World Health Organization (WHO) ASSURED (‘Affordable, Sensitive, Specific, User-friendly, Rapid and robust, Equipment-free, and Deliverable to end users’) criteria and may be more suitable for use in resource-constrained settings. Several evaluation studies or clinical use of the LFA have reported a general high analytical performance and strong clinical relevance.^15–19^

In this study, we screened for CPA among patients presumed to have PTB using the LDBio *Aspergillus*-specific IgG and IgM LFA and the CPA guideline for resource-constrained settings.^8^ We also evaluated the significance of CPA as a differential diagnosis of PTB and assessed the clinical relevance of the LDBio *Aspergillus*-specific IgG and IgM LFA in CPA diagnosis.

## Materials and methods

### Study design and site

The study was a cross-sectional survey conducted at the Chest Clinic, Chest Diseases Unit, Department of Medicine and Therapeutics, Korle-Bu Teaching Hospital, Accra. The clinic acts as the national TB referral centre and hosts a specialised TB laboratory that receives samples from different parts of Ghana.

### Study population

Patients seen at the Chest Clinic of the Korle-Bu Teaching Hospital for suspected PTB or those referred from other parts of the country to the TB laboratory for GeneXpert MTB (Xpert^®^ MTB/RIF, Cepheid, CA, USA) testing were recruited, irrespective of their symptoms. Also, blood donors determined to have no symptoms and signs of a respiratory condition or history of PTB, or any other chronic respiratory condition via an interview, were also recruited as control group participants from the National Blood Service, Ghana. This was mainly done to assess the specificity of the LFA test.

Ethical approval was obtained from the Institutional Review Board of the Korle-Bu Teaching Hospital (STC/IRB/00058/2020) and the National Blood Services Ghana (NBSGRD/201410/02) and University Research and Ethics Committee of the University of Manchester (Ref: 2020-9368-16168). Written informed consent was obtained from all participants.

### Investigations

Patients’ demographics, clinical and socioeconomic details were collected via interviews using a questionnaire. Serum samples were obtained for *Aspergillus* serology with LDBio *Aspergillus* IgG and IgM LFA (LDBio Diagnostics, Lyon, France) and HIV antibody testing with HIV ½ RDT (Healgen Scientific LLC, Houston, TX, USA) and confirmed with OraQuick HIV ½ RDT (OraSure Technologies, Bethlehem, PA, USA). Sputum was obtained for high-volume culture to enhance *Aspergillus* detection, a modified version of Vergidis et al.,^20^ by inoculating an aliquot (1–2 ml) of undiluted specimen on Sabouraud dextrose agar and incubated at 37°C for up to 8 days. Chest radiograph was done for all patients unless patient had obtained one within the previous month. Xpert MTB/RIF results were retrieved from laboratory records. Chest computed tomography (CT) scan was done for patients with positive *Aspergillus* serology or cavitation on chest radiograph including MTB-positive cases. Among the MTB-positive cases with cavitation, CT scan was done to unravel any concealed imaging features of CPA to identify possible PTB-CPA coinfection. In the control group, only serum samples were obtained for *Aspergillus* serology.

### Case definition

TB was diagnosed if MTB was detected in a patient's sputum by Xpert MTB/RIF assay. Patients with suspected or confirmed PTB were classified as new PTB and relapsed PTB as follows:

- new PTB; patients with no prior history of PTB
- relapsed PTB; patients who had been treated successfully for PTB in the past

A case of CPA was defined following the guidelines for CPA diagnosis in resource-constrained settings, developed by the GAFFI international expert panel (2018).^8^ The panel defined a case of CPA as follows:

- weight loss, persistent cough, and/or haemoptysis for >3 months
- chest images showing progressive cavitary infiltrates and/or a fungal ball and/or pericavitary fibrosis or infiltrates or pleural thickening
- positive *Aspergillus* IgG assay or other evidence of *Aspergillus* infection.

Patients who met criteria (i) and (ii) above, but not (iii), or met criteria (i) and (iii), but not (ii) were categorised as probable CPA, a modified version of a classification described by Setianingrum et al.^21^

### Data analysis

Data were analysed with SPSS version 25 (IBM, Armonk, NY, USA) at 5% significance level, using either Chi-square or Fisher's exact tests. Summary statistics were presented using frequencies and percentages for categorical variables, and median values for non-normally distributed continuous variables. Fisher's exact tests were employed to compare proportions between groups. Logistic regression was carried out to assess the effect of individual symptoms and socioeconomic details on the likelihood of acquiring CPA.

## Results

From October 2020 to May 2021, 183 consecutive patients referred for Xpert MTB/RIF were screened, but 21 (11.5%) were either less than 18 years or unable to provide sputum and/or blood and were excluded. Of the 162 recruited, a complete data set for evaluation of CPA was available for 154 (84.2%) patients. The 154 patients comprised 92 (59.7%) males, with a median age of 41.5 years and range of 18 to 96 years (Table 1). There were 134 (87%) and 20 (13%) patients being assessed for new PTB and ‘relapsed’ PTB, respectively. The time from completion of TB treatment to recruitment in the ‘relapse’ group was 1 to 24 years (median 4). The median duration of symptoms prior to presentation among patients was 9 weeks, with a range of 1 to 21 weeks. Ninety blood donors were recruited in the control group from March to April 2021.

**Table 1.** Characteristics of 154 patients referred for GeneXpert TB according to eventual CPA diagnosis.

Features	Total (n = 154)	CPA (n = 15)	Non-CPA (n = 139)	*P* value
Demographics				
Male	92 (59.7%)	11 (73.3%)	81 (58.3%)	
Female	62 (40.3%)	4 (26.7%)	58 (41.7%)	.410
Age, median (range)	41.5 (18-96)	47 (28-96)	43.4 (18-78)	.765
Clinical details				
History of previous PTB	20 (13%)	10 (66.7%)	10 (7.2%)	.002
Persistent cough	138 (89.6%)	15 (100%)	123 (88.5%)	1.0
Haemoptysis	26 (16.9%)	7 (46.7%)	19 (13.7%)	.023
Chest pain	74 (48.1%)	11 (73.3%)	63 (45.3%)	.395
Dyspnoea	57 (37%)	4 (26.7%)	53 (38.1%)	.570
Fatigue	111(72.1%)	11 (73.3%)	100 (71.9%)	.101
Weight loss	110 (71.4%)	10 (66.7%)	100 (71.9%)	.203
Chronic condition				
Asthma	6 (3.9%)	2 (13.3%)	4 (2.9%)	.040
COPD	9 (5.8%)	3 (20%)	6 (4.3%)	.044
Diabetes mellitus	7 (4.5%)	1 (6.7%)	6 (4.3%)	.500
Hypertension	25 (16.2%)	3 (20%)	22 (15.8%)	.101
Lung cancer	1 (0.6%)	0	1 (0.7%)	1.0
Socioeconomics				
Practice of traditional cooking^a^	83 (53.9%)	7 (46.7%)	76 (54.7%)	.433
Residence in damp house	12 (7.8%)	2 (13.3%)	10 (7.2%)	.330
Engagement in agricultural activities	30 (19.5%)	2 (13.3%)	28 (20.1%)	.740
History of smoking	27 (17.5%)	2 (13.3%)	25 (18%)	1.0

a Cooking with charcoal or firewood.

### Laboratory results

Laboratory findings of the 154 patients are shown in Table 2. MTB was detected in 41 (26.6%) patients, of whom 35 (85.4%) were classified as new PTB and 6 (14.6%) had relapsed PTB. *Aspergillus* serology was positive in 9.9% (*n* = 16) of participants, but there was no imaging data for two (12.5%) of these and hence they were excluded from the analysis. There were 44 (28.6%) HIV-positive patients, of whom 2 (4.5%) had positive *Aspergillus* serology. *Aspergillus* serology was positive in one (1.1%) participant in the control group, who had no respiratory signs and symptoms or no previous history of PTB. Culture was positive for *Aspergillus* spp. in 32 (20.8%) cases, yielding 38 isolates. The main species were *Aspergillus fumigatus* (47.4%, *n* = 18) and *A. niger* (36.8%, *n* = 14) (Table 2).

**Table 2.** Laboratory results of 154 patients referred for GeneXpert TB according to eventual CPA diagnosis.

Variable	Total (n = 154)	CPA (n = 15)	Non-CPA (n = 139)	*P* value
*Aspergillus* serology	14 (9.1%)	14 (93.3%)	0	<.001
Positive *Aspergillus* culture	32 (20.8%)	10 (66.7%)	21 (15.1%)	.024
*Aspergillus* spp. isolates	38 (24.7%)	13 (86.7%)	25 (18.0)	<.001
*Aspergillus* spp. distribution				
*Aspergillus fumigatus*	18 (47.4%)	9 (69.2%)	9 (36%)	
*Aspergillus niger*	14 (36.8%)	3 (23.1%)	11 (44%)	
*Aspergillus flavus*	5 (13.2%)	1 (7.7%)	4 (16%)	
*Aspergillus terreus*	1 (2.6%)	0	1 (4%)	
HIV reactive	44 (28.6%)	3 (20%)	41 (29.5%)	.560
MTB detected	41 (26.6%)	4 (26.7%)	37 (26.6%)	1.0
MTB load distribution				
Trace	2 (4.9%)	2 (50.0%)	0	.009
Very low	5 (12.2%)	2 (50.0%)	3 (8.1%)	.050
Low	3 (7.3%)	0	3 (8.1%)	1.0
Medium	9 (22%)	0	9 (24.3%)	.060
High	22 (53.7%)	0	22 (59.5%)	.013

### Radiological findings

**Figure 1.**
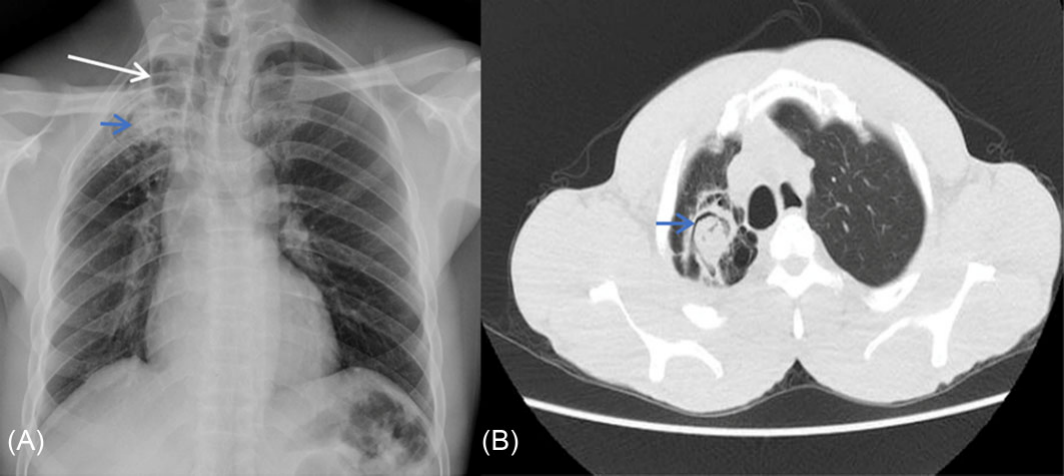
(A) Plain frontal radiograph of a CPA patient shows a right apical lung cavity (long arrow) with a soft tissue density within it (short arrow), which is associated with pericavity fibrosis and volume loss evidenced by mediastinal shift to the right. (B) Axial chest CT scan in lung window confirmed the presence of a right apical lung aspergilloma with an air crescent sign (short arrow) and surrounding fibrosis 164 × 73 mm (144 × 144 DPI).

**Figure 2.**
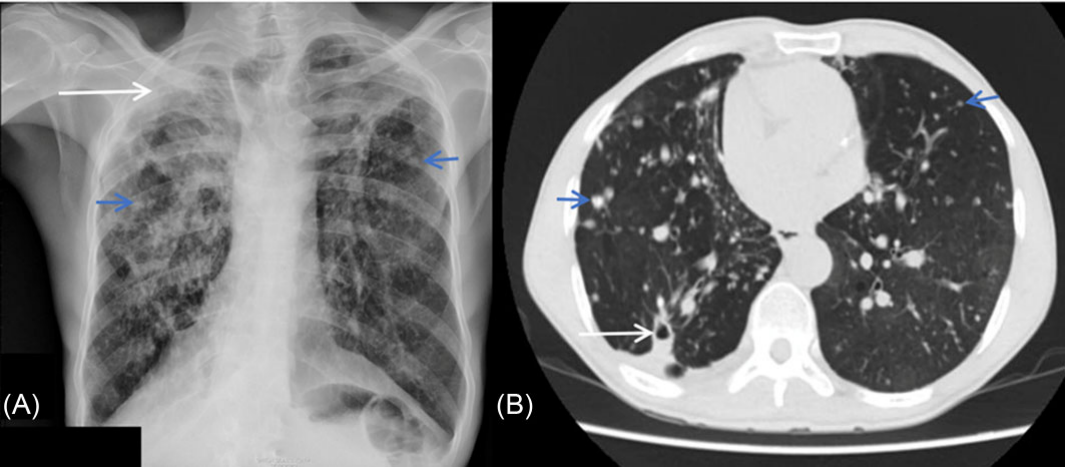
(A) Plain frontal radiograph of another CPA patient demonstrates bilateral apical lung fibrosis with associated hilar retraction and distortion. Right apical pleural thickening (long arrow) and adjacent consolidation. Background of diffuse bilateral lung nodules (short arrows). (B) Corresponding axial chest CT scan in lung window demonstrates a small right lower lobe superior segment cavity, which was not demonstrated on the plain radiograph and multiple bilateral lung nodules (short arrows) 159 × 70 mm (144 × 144 DPI).

Of 154 patients, chest radiograph was normal in 96 (62.3%) (Table 3). The common abnormalities reported were infiltration (23.4%, *n* = 36), cavitation (16.9%, *n* = 26), fibrosis (12.3%, *n* = 19), and pleural thickening (15.6%, *n* = 24). Chest CT scan was done in 17 (53.1%) of the 32 patients eligible for the procedure; the remainder either died or were lost to follow-up. The major CT scan findings were cavitation (100%, *n* = 17; two of these contained a fungal ball), fibrosis (88.2%, *n* = 15), and pleural thickening (88.2%, *n* = 15). Out of the 26 participants with cavitation, 11 (42.3%) had a positive *Aspergillus* IgG/IgM assay. Cavitation (*P* = .005), paracavitary fibrosis (*P* = .005), and pleural thickening (*P* = .04) were seen more often in patients with CPA (Table 3). CT scan contributed to CPA diagnosis in 11 patients (Figs 1 and 2).

**Table 3.** Imaging (chest radiograph and/or CT scan) findings for 154 patients.

Variable	Total (n = 154)	CPA (n = 15)	Non-CPA (n = 139)	*P* value
Infiltration	36 (23.4%)	8 (53.3%)	28 (20.1%)	.169
Cavitation	26 (16.9%)	12 (80%)	14 (10.1%)	.005
Paracavitary fibrosis	19 (12.3%)	11 (73.3%)	8 (5.8%)	.005
Pleural thickening	24 (15.6%)	10 (66.7%)	14 (10.1%)	.040
Bronchiectasis	11 (7.1%)	3 (20%)	8 (5.8%)	.077
Nodules	16 (10.4%)	2 (13.3%)	14 (10.1%)	.066
Fungal ball	2 (1.3%)	2 (13.3%)	0	.009
Pleural effusion	12 (7.8%)	1 (6.7%)	11 (7.9%)	1.0

### CPA classification

Of the 154 patients, 15 (9.7%, 95% CI 7.8%–11.6%) met the criteria for CPA, including three probable CPA cases (Supplementary Table S1). Ten (66.7%) CPA cases had previous PTB, representing 50% (10/20) of the patients being assessed for relapsed PTB. The predisposing conditions in the remaining CPA patients were chronic obstructive pulmonary disease (COPD) (*n* = 3) and asthma (*n* = 2). Four cases classified as CPA, all with previous history of PTB infection, had trace or very low levels of MTB detected on GeneXpert, and were placed on TB treatment. The diagnosis of CPA was made in these patients because the imaging features were more suggestive of CPA including cavitation with irregular intraluminal lining of cavity, pleural thickening adjacent cavity, paracavitary fibrosis, and one with a fungal ball. Additionally, *Aspergillus* antibody test was positive in all four patients and *Aspergillus* spp. was isolated in three patients. Three subsequently had a negative sputum Xpert MTB and/or AFB smear 1 month later but the fourth patient had a positive AFB smear which showed scanty organisms. The proportion of CPA based on HIV status were 10.9% (12/110) and 6.8% (3/44) for HIV-negative and HIV-positive, respectively (*P* = .560). The most common symptoms among CPA cases were fatigue (73.3%, *n* = 11), weight loss (73.3%, *n* = 11), and haemoptysis (46.7%, *n* = 7).

Haemoptysis was proportionately more common in CPA than other symptoms. No association was found between CPA diagnosis and socioeconomic details as potential risk factors.

## Discussion

This study is the first epidemiological study on CPA from Ghana and provides data for differential PTB diagnosis. It is common practice in high TB prevalence areas that patients previously treated for PTB who present with new symptoms are generally diagnosed with PTB relapse even when sputum AFB smear or Xpert MTB are negative. Our study showed that half of these patients had CPA. Therefore, a prior history of PTB treatment in a patient presenting with suspected relapse should raise suspicion for CPA.

The current study revealed a CPA prevalence of 9.7% among patients presenting with presumed PTB. Varying rates have also reported in Uganda^22^ and Nigeria,^6^ Iran,^23^ Pakistan,^24^ Indonesia,^15,21^ Uganda,^16^ and Brazil^25^ largely due to difference in study designs, population, investigations, and sampling methods. Also, the present study identified 50% prevalence of CPA in patients with prior PTB. This is similar to recent reports from Vietnam (54.3%)^26^ and India (57%).^27^ CPA and PTB often present with clinically indistinguishable symptoms; fever is more common in PTB, and haemoptysis in CPA, but are not sufficiently distinctive features to be used as a definite diagnostic tool. A recent review established a common association between CPA and TB in Africa.^28^ Considering the high burden of TB in many African countries such as Ghana, CPA is likely to be misdiagnosed as PTB.^5^ In recent times, the new GAFFI case definition for CPA for resource-constrained healthcare settings, utilising the new *Aspergillus* IgG and IgM LFA, is improving epidemiological studies and providing more clinical experience of CPA in resource-constrained settings.^7,15–19^

In the present study, three of four patients with concomitant CPA and PTB had a negative AFB smear within a month. The average interval from completion of TB treatment to recruitment for these patients was 4 years. Possibly, their Xpert MTB could have been false positives due to detection of residual MTB DNA or non-viable non-intact bacilli.^29–31^ The role of Xpert MTB in detecting reinfection or relapse accurately is unclear, often associated with high occurrence of false positives especially with low mycobacterial burden, and short time post-treatment, as noted in our study.^30,31^ The likelihood of false positivity is reported to decrease with the longer time since successful treatment of PTB but the total duration is not known.^30^ Nevertheless, one study indicates that this can be up to 4 years after successful completion of appropriate treatment.^31^ The fourth patient had a positive AFB smear result, and probably had PTB-CPA co-infection.

The profound drug–drug interaction between rifampicin and oral antifungal azoles requires that clinicians to select between these diagnoses, and not attempt treating both PTB and CPA together. Some very ill patients with CPA require intravenous antifungal therapy while those with a single aspergilloma can be cured with surgical resection.^4^ However, the majority require at least 12 months of oral antifungal (itraconazole or voriconazole) treatment for chronic cavitary pulmonary aspergillosis to reduce symptoms, prevent progression or relapse and improve overall quality of life.^4^ A recent randomised controlled trial in India demonstrated that 12 months of oral itraconazole was superior to a 6-month regimen in reducing relapses of CPA at 2 years.^32^

The most common symptoms for CPA cases recorded in the current study were fatigue, weight loss, and haemoptysis. Although patients with active PTB also had these symptoms, haemoptysis was more common in CPA, and should raise suspicion for diagnosis.^5^ COPD was more common in patients with CPA than PTB, although overall numbers were small. We observed no significant statistical difference in the rate of CPA in HIV-positive patients and CPA in HIV-negative patients (6.8% vs 10.9%), contrary to other studies.^6,16,22,33^ Though *Aspergillus* serology was positive in less than 10% of our screened patients, it contributed to greater than 90% of CPA diagnosis. One CPA patient who was HIV-positive had a negative *Aspergillus* IgG and IgM LFA result. This maybe because of a reduced capacity to elicit production of antibodies; the correlation between immunodeficiency and negative *Aspergillus* serology has been previously described.^6,9,33^ Unfortunately, the participant in the control group with a positive *Aspergillus* antibody test was unable to be reached for imaging and further laboratory investigations to evaluate the clinical relevance. It could have been a possible case of asymptomatic pulmonary aspergillosis or a false positive test. In addition, we did not observe an association with various sociodemographic practices common in Ghana that could potentially lead to fungal exposure.

The most common organism implicated in CPA is *A. fumigatus*; it is especially reported in Europe and United States, where it accounts for over 90% of all cases.^34–36^ However, in Africa and Asia, *A. flavus* and *A. niger* are frequently isolated, as demonstrated in our study and elsewhere.^6,15,16,34^ The frequent imaging findings of CPA, as stipulated in many guidelines, reported in several epidemiological studies, and considered to be more linked to CPA are cavitation, pericavitary fibrosis, and pleural thickening.^4,5,8,15,21,22^ These three features were present in 67% – 80% of CPA cases in our study on chest radiograph and/or CT scan. Three patients without cavitation on chest radiograph were diagnosed with probable CPA based on parenchymal fibrosis and/or bronchiectasis coupled with positive *Aspergillus* serology and/or culture. It is possible that these patients had cavitation that could have been revealed by CT scan as observed in two other patients with CPA. CT scan is an important complementary investigation to chest radiography when available especially when *Aspergillus* serology is positive. In the present study, additional cavitation and fungal balls detected by CT scan in four patients were missed on chest radiographs. Similar observations were made by Page et al.^22^ and Nguyen et al.^26^

In conclusion, the early differentiation of active PTB, post-TB lung disease, and PTB plus CPA co-infection, in settings with high TB burden, may require a broader screening strategy at the investigation stage. The present study contributes to the efforts of identifying an efficient framework for routine or systematic screening for CPA in PTB. Access to readily available diagnostics, in addition to algorithms that easily identify patients with CPA, will improve patient care and outcomes.

Our study is not without limitations. Our inability to do CT scans for all eligible patients due to loss to follow-up or death was a major challenge. Secondly, prior to PTB retreatment, a repeat Xpert MTB and/or culture was not done for CPA patients from whom MTB was detected to rule out false positive results. Additionally, patients being assessed for PTB relapse were few, and the CPA rates in this group may not be representative.

## Supplementary Material

myac063_Supplemental_File

## References

[1] Ekeng BE , Davies AA, Osaigbovo II, Warris A, Oladele RO, Denning DW. Pulmonary and extrapulmonary manifestations of fungal infections misdiagnosed as tuberculosis: the need for prompt diagnosis and management. J Fungi. 2022; 8: 460. 10.3390/jof8050460 PMC9143176 35628715

[2] Denning DW , Pleuvry A, Cole DC. Global burden of chronic pulmonary aspergillosis as a sequel to pulmonary tuberculosis. Bull World Health Organ. 2011; 89: 864–872. 22271943 10.2471/BLT.11.089441 PMC3260898

[3] Ocansey BK , Pesewu GA, Codjoe FS, Osei-Djarbeng S, Feglo PK, Denning DW. Estimated burden of serious fungal infections in Ghana. J Fungi (Basel). 2019; 5: 38. 10.3390/jof5020038 PMC6616901 31083531

[4] Denning DW , Cadranel J, Beigelman-Aubry C et al. Chronic pulmonary aspergillosis: rationale and clinical guidelines for diagnosis and management. Eur Respir J. 2016; 47: 45–68. 26699723 10.1183/13993003.00583-2015

[5] Baluku JB , Nuwagira E, Bongomin F, Denning DW. Pulmonary TB and chronic pulmonary aspergillosis: clinical differences and similarities. Int J Tuberc Lung Dis. 2021; 25: 537–546. 34183098 10.5588/ijtld.21.0034

[6] Oladele RO , Irurhe NK, Foden P et al. Chronic pulmonary aspergillosis as a cause of smear-negative TB and/or TB treatment failure in Nigerians. Int J Tuberc Lung Dis. 2017; 21: 1056–1061. 28826456 10.5588/ijtld.17.0060

[7] Kwizera R , Katende A, Bongomin F, Nakiyingi L, Kirenga BJ. Misdiagnosis of chronic pulmonary aspergillosis as pulmonary tuberculosis at a tertiary care center in Uganda: a case series. J Med Case Rep. 2021; 15: 140. 33781313 10.1186/s13256-021-02721-9 PMC8007227

[8] Denning DW , Page ID, Chakaya J et al. Case definition of chronic pulmonary aspergillosis in resource-constrained settings. Emerg Infect Dis. 2018; 24: e171312. 10.3201/eid2408.171312 PMC6056117 30016256

[9] Page ID , Richardson M, Denning DW. Antibody testing in aspergillosis–quo vadis? Med Mycol. 2015; 53: 417–439. 25980000 10.1093/mmy/myv020

[10] Li H , Rui Y, Zhou W et al. Role of the aspergillus-specific IgG and IgM test in the diagnosis and follow-up of chronic pulmonary aspergillosis. Front Microbiol. 2019; 10. Accessed April 29, 2022. https://www.frontiersin.org/article/10.3389/fmicb.2019.01438 10.3389/fmicb.2019.01438 PMC6611396 31316486

[11] Anan K , Kataoka Y, Okabayashi S, Yamamoto R, Namkoong H, Yamamoto Y. Diagnostic accuracy of Aspergillus-specific antibodies for chronic pulmonary aspergillosis: a systematic review and meta-analysis. Mycoses. 2021; 64: 701–715. 33594774 10.1111/myc.13253

[12] Richardson MD , Page ID. Aspergillus serology: have we arrived yet? Med Mycol. 2017; 55: 48–55. 27816904 10.1093/mmy/myw116

[13] Page ID , Richardson MD, Denning DW. Comparison of six Aspergillus-specific IgG assays for the diagnosis of chronic pulmonary aspergillosis (CPA). J Infect. 2016; 72: 240–249. 26680697 10.1016/j.jinf.2015.11.003

[14] Sehgal IS , Choudhary H, Dhooria S et al. Diagnostic cut-off of Aspergillus fumigatus-specific IgG in the diagnosis of chronic pulmonary aspergillosis. Mycoses. 2018; 61: 770–776. 29920796 10.1111/myc.12815

[15] Rozaliyani A , Rosianawati H, Handayani D et al. Chronic pulmonary aspergillosis in post tuberculosis patients in indonesia and the role of LDBio Aspergillus ICT as part of the diagnosis scheme. J Fungi. 2020; 6: 318. 10.3390/jof6040318 PMC7712371 33260909

[16] Namusobya M , Bongomin F, Mukisa J et al. Chronic pulmonary aspergillosis in patients with active pulmonary tuberculosis with persisting symptoms in Uganda. Mycoses. 2022; 65: 625–634. 35419885 10.1111/myc.13444 PMC9156563

[17] Kwizera R , Katende A, Teu A et al. Algorithm-aided diagnosis of chronic pulmonary aspergillosis in low- and middle-income countries by use of a lateral flow device. Eur J Clin Microbiol Infect Dis. 2020; 39: 1–3. 31811506 10.1007/s10096-019-03782-x

[18] Ray A , Chowdhury M, Sachdev J et al. Efficacy of LDBio Aspergillus ICT lateral flow assay for serodiagnosis of chronic pulmonary aspergillosis. J Fungi (Basel). 2022; 8: 400. 35448631 10.3390/jof8040400 PMC9029852

[19] Singh S , Choudhary H, Agnihotri S et al. LDBio Aspergillus immunochromatographic test lateral flow assay for IgG/IgM antibody detection in chronic pulmonary aspergillosis: single-centre evaluation and meta-analysis. Indian J Med Microbiol. 2022; 40: 204–210. 35370006 10.1016/j.ijmmb.2022.03.002

[20] Vergidis P , Moore CB, Novak-Frazer L et al. High-volume culture and quantitative real-time PCR for the detection of Aspergillus in sputum. Clin Microbiol Infect. 2020; 26: 935–940. 31811917 10.1016/j.cmi.2019.11.019

[21] Setianingrum F , Rozaliyani A, Adawiyah R et al. A prospective longitudinal study of chronic pulmonary aspergillosis in pulmonary tuberculosis in Indonesia (APICAL). Thorax. 2022; 77: 821–828. 34848556 10.1136/thoraxjnl-2020-216464 PMC9340040

[22] Page ID , Byanyima R, Hosmane S et al. Chronic pulmonary aspergillosis commonly complicates treated pulmonary tuberculosis with residual cavitation. Eur Respir J. 2019; 53: 1801184. 30705126 10.1183/13993003.01184-2018 PMC6422837

[23] Hedayati MT , Azimi Y, Droudinia A et al. Prevalence of chronic pulmonary aspergillosis in patients with tuberculosis from Iran. Eur J Clin Microbiol Infect Dis. 2015; 34: 1759–1765. 26003310 10.1007/s10096-015-2409-7

[24] Zubair SM , Jabeen K, Irfan M. Frequency of chronic pulmonary aspergillosis in patients treated for pulmonary tuberculosis at a tertiary care hospital in Karachi, Pakistan. Eur Respir J. 2021; 58: PA1024. 10.1183/13993003.congress-2021.PA1024

[25] Volpe-Chaves CE , Venturini J, Castilho S B et al. Prevalence of chronic pulmonary aspergillosis regarding time of tuberculosis diagnosis in Brazil. Mycoses. 2022; 65: 715–723. 35524507 10.1111/myc.13465

[26] Nguyen NTB , Le Ngoc H, Nguyen NV et al. Chronic pulmonary aspergillosis situation among post tuberculosis patients in Vietnam: an observational study. J Fungi (Basel). 2021; 7: 532. 34209322 10.3390/jof7070532 PMC8307285

[27] Singla R , Singhal R, Rathore R et al. Risk factors for chronic pulmonary aspergillosis in post-TB patients. Int J Tuberc Lung Dis. 2021; 25: 324–326. 33762078 10.5588/ijtld.20.0735

[28] Olum R , Osaigbovo II, Baluku JB, Stemler J, Kwizera R, Bongomin F. Mapping of chronic pulmonary aspergillosis in Africa. J Fungi (Basel). 2021; 7: 790. 34682212 10.3390/jof7100790 PMC8541146

[29] Costantini L , Marando M, Gianella P. Long-Term GeneXpert positivity after treatment for pulmonary tuberculosis. Eur J Case Rep Intern Med. 2020; 7: 001737. 33083351 10.12890/2020_001737 PMC7546557

[30] Theron G , Venter R, Smith L et al. False-Positive Xpert MTB/RIF results in retested patients with previous tuberculosis: frequency, profile, and prospective clinical outcomes. J Clin Microbiol. 2018; 56: e01696–17. 29305538 10.1128/JCM.01696-17 PMC5824043

[31] Theron G , Venter R, Calligaro G et al. Xpert MTB/RIF results in patients with previous tuberculosis: can we distinguish true from false positive results? Clin Infect Dis. 2016; 62: 995–1001. 26908793 10.1093/cid/civ1223 PMC4803105

[32] Sehgal IS , Dhooria S, Muthu V et al. Efficacy of 12-months oral itraconazole versus 6-months oral itraconazole to prevent relapses of chronic pulmonary aspergillosis: an open-label, randomised controlled trial in India. Lancet Infect Dis. 2022; 22: 1052–1061. 35429465 10.1016/S1473-3099(22)00057-3

[33] Hunter ES , Wilopo B, Richardson MD, Kosmidis C, Denning DW. Effect of patient immunodeficiencies on the diagnostic performance of serological assays to detect Aspergillus-specific antibodies in chronic pulmonary aspergillosis. Respir Med. 2021; 178: 106290. 33529993 10.1016/j.rmed.2020.106290 PMC7957343

[34] Barac A , Kosmidis C, Alastruey-Izquierdo A, Salzer HJF. Chronic pulmonary aspergillosis update: a year in review. Med Mycol. 2019; 57: S104–S109. 30816975 10.1093/mmy/myy070

[35] Kosmidis C , Denning DW. The clinical spectrum of pulmonary aspergillosis. Thorax. 2015; 70: 270–277. 25354514 10.1136/thoraxjnl-2014-206291

[36] Pena TA , Soubani AO, Samavati L. Aspergillus lung disease in patients with sarcoidosis: a case series and review of the literature. Lung. 2011; 189: 167–172. 21327836 10.1007/s00408-011-9280-9

