# Supplementary material for: Chronic pulmonary aspergillosis is common among patients with presumed tuberculosis relapse in Ghana
Source: Med Mycol. 2022 Aug 11;60(9):myac063. doi: 10.1093/mmy/myac063 (PMC9462665; doi:10.1093/mmy/myac063)
Supplement: myac063_Supplemental_File [file myac063_supplemental_file.docx]

| **SNo** | **Sex** | **Age** | **Previous PTB** | **Xpert MTB** | **HIV** | **ASPG LFA** | **Culture** | **Relevant CXR and/or CT scan findings** |
| --- | --- | --- | --- | --- | --- | --- | --- | --- |
| 1 | M | 43 | Yes | Pos-Very low | Neg | Pos | Neg | Cavitation, fibrosis, infiltration, pleural thickening, nodules |
| 2 | F | 33 | Yes | Neg | Neg | Pos | A. *fumigatus* | Cavitation, pleural thickening, bronchiectasis, hydropneumothorax |
| 3 | F | 52 | Yes | Neg | Neg | Pos | Neg | Fibrosis, infiltration, pleural thickening, hydropneumothorax |
| 4 | M | 33 | Yes | Pos-Trace | Neg | Pos | A. *fumigatus*, A. *niger* | Cavitation, fungal ball, fibrosis, pleural thickening, |
| 5 | M | 30 | No | Neg | Neg | Pos | A. *fumigatus* | Cavitation, fibrosis, infiltration, pleural thickening |
| 6 | M | 73 | Yes | Neg | Neg | Pos | A. *fumigatus*, A. *niger* | Cavitation, fibrosis, infiltration, pleural thickening, bronchiectasis |
| 7 | M | 53 | Yes | Pos-Trace | Neg | Pos | A. *fumigatus* | Cavitation, fibrosis, infiltration, pleural thickening, bronchiectasis |
| 8 | F | 45 | No | Neg | Pos | Pos | A. *fumigatus*, A. *flavus* | Fibrosis, bronchiectasis |
| 9 | M | 72 | No | Neg | Neg | Pos | A. *fumigatus*, A. *niger* | Cavitation, pleural thickening, fibrosis |
| 10 | M | 96 | No | Neg | Pos | Pos | Neg | Fibrosis, bronchiectasis |
| 11 | M | 62 | Yes | Neg | Neg | Pos | A. *fumigatus* | Cavitation, fibrosis, pleural thickening, pleural effusion |
| 12 | M | 28 | Yes | Neg | Neg | Pos | Neg | Cavitation, fibrosis, infiltration, pleural thickening |
| 13 | M | 47 | Yes | Neg | Neg | Pos | Neg | Cavitation, fibrosis, |
| 14 | M | 51 | Yes | Pos-Very low | Neg | Pos | A. *flavus* | Cavitation, fibrosis, pleural thickening, bronchiectasis, nodules |
| 15 | F | 42 | No | Neg | Pos | Neg | A. *fumigatus* | Cavitation, infiltration, pleural thickening |

**Table S1: Demographic, clinical details, laboratory and imaging findings of CPA patients**
